# Evaluation of the responses of contrasting sensitive and tolerant rice genotypes to arsenic stress

**DOI:** 10.1007/s44154-024-00185-7

**Published:** 2025-03-05

**Authors:** Poonam Yadav, Meghna Jaiswal, Sudhakar Srivastava

**Affiliations:** Plant Stress Biology Laboratory, Institute of Environment and Sustainable Development, Banaras Hindu University, Varanasi, 221005 India

**Keywords:** Arsenic, Germination, Non-protein thiol, Rice variety, Seedling vigour, Superoxide dismutase

## Abstract

The threats of arsenic (As) contamination of rice grains and rice-based food products are being experienced globally. Arsenic toxicity to rice (*Oryza sativa L.*) affects its production and grain quality. In this work, screening of 67 rice genotypes was performed against As stress (25 µM and 50 µM, in the form of arsenite [As(III)]) for 7d in lab conditions on the basis of germination and early growth of seedlings. Germination percentage, root and shoot length, and seedling vigour were analyzed. The genotypes showed a wide range of variation in germination percentage, and seedling growth in response to both control and As(III) stress. The comparative evaluation of genotypes could demarcate them into sensitive (e.g. Shobhini and DRR Dhan-41) and tolerant (e.g. Pooja and Vivek Dhan) categories. To further investigate the behavior of contrasting varieties, a tolerant (Pooja) and moderately sensitive (CO-50) variety of rice were subjected to As stress for variable concentrations (0–25 µM; fixed duration 5 d) and duration (1–15 d; fixed concentration 25 µM). The accumulation of As by tolerant and sensitive varieties in roots, old leaves and young leaves was found to be significantly lower in Pooja than in CO-50. The differential As accumulation reflected in growth and biochemical responses (malondialdehyde level, superoxide dismutase activity and total non-protein thiols). The results demonstrate the potential of using contrasting rice varieties for future breeding or gene editing approaches to develop low arsenic accumulating rice cultivars.

**Supplementary Information:**

The online version contains supplementary material available at 10.1007/s44154-024-00185-7.

## Introduction

Arsenic (As) is an element popularly known as the king of poisons (Bhowmick et al. 2018). Its contamination of groundwater and soil is a serious and widespread problem affecting the health of nearly 150 million people worldwide (Brammer and Ravenscroft 2009). The condition of As contamination of soil and groundwater is severe in the Bengal delta and Bangladesh and is regarded as the greatest mass poisoning in human history (Smith et al. 2000; Chakraborti et al. 2004). In contaminated areas, As concentration in groundwater exceeds the safe limit (10 µg L^−1^) defined by the World Health Organization. The groundwater is regularly used for irrigation of crops including rice, which is a semi-aquatic plants requiring submerged conditions for optimum growth (Awasthi et al. 2017). The anaerobic conditions generated by submerged irrigation of rice cultivation, and the presence of As mostly in the inorganic arsenite [As(III)] form in these conditions results in As accumulation in rice grains in greater quantity than that in any other crop plant (Awasthi et al. 2017). Rice grains from Bangladesh and West Bengal, India have been found to contain higher than safe and recommended As levels (Upadhyay et al. 2019). Further, the presence of higher than safe As levels has also been detected in rice based food products, including baby foods (Upadhyay et al. 2019). Rice is the main staple food crop in Southeast Asian countries consuming approximately 300 g of rice per day (Muthayya et al. 2014). Therefore, rice acts as an important source of As for people and hence, As accumulation in rice needs to be managed.

The alternatives to tackle the “As in rice” problem include (1) removal of As from groundwater and soil, which is not a practical and feasible approach to tackle such a large scale problem and (2) to restrict As entry into rice through agronomic or biotechnological approaches. The second option is feasible and a number of strategies, comprising of fertilizer /elemental/microbial algal supplementations, are being tested at lab or field scale (Srivastava et al. 2016; Kumar et al. 2016; Seyfferth et al. 2017; Awasthi et al. 2018). Another practical and applicable way to tackle the problem is the screening and selection of suitable rice genotypes that naturally resist As entry into their grains and show good yields in As contaminated soils. Thus, locally adaptable appropriate rice genotypes could be a feasible and a low cost approach. To this end, a few studies have focused on screening and identification of low As accumulating or As tolerant rice varieties and genotypes (Norton et al. 2009; Pillai et al. 2010; Ahmed et al. 2011). A significant variation in grain total As concentration and As species levels was found among different genotypes. In India, Dwivedi et al. (2012) conducted field trial of 90 rice genotypes in West Bengal, India and reported significant variation in total grain As concentration in these genotypes. Dave et al. (2013) screened 303 genotypes of rice in laboratory conditions against As(III) stress and identified As tolerant and As sensitive varieties. Majumder et al. (2018) screened eight rice cultivars for As tolerance by hydroponic cultivation in arsenate [As(V)] containing medium. They could identify Kumargore, Binni, Vijaya and Bhutmuri as suitable As tolerant cultivars and recommended the cultivation of these cultivars in As contaminated areas.

The symptoms of As toxicity can be witnessed as poor seed germination and early seedling growth (Abedin et al. 2002; Srivastava et al. 2013a), photosynthetic and redox status disturbance and stimulation of antioxidant status and thiol metabolism (Srivastava et al. 2013b; Awasthi et al. 2017). Dasgupta et al. (2004) screened 20 rice genotypes on the basis of root growth reduction against 13.3 µM As(V) stress and reported marked differences in tolerance of different varieties. While CO39 variety was most tolerant to 13.3 µMAs(V), Dawn was the most sensitive variety. Considering the suitability of germination and early seedling growth based screening (Dave et al. 2013), the present study was conducted to screen sixty seven (67) rice varieties for As tolerance so as to identify potential groups of tolerance and sensitive varieties. Further, the study also assessed the concentration- and duration-dependent dependent responses of contrasting genotypes to As stress with respect to As accumulation, growth and biochemical responses. The results demonstrate the potential benefits of using tolerant rice genotype for cultivation in As-contaminated areas.

## Results and discussion

### Rice genotype screening and identification of tolerant and sensitive genotype

**Fig. 1:**
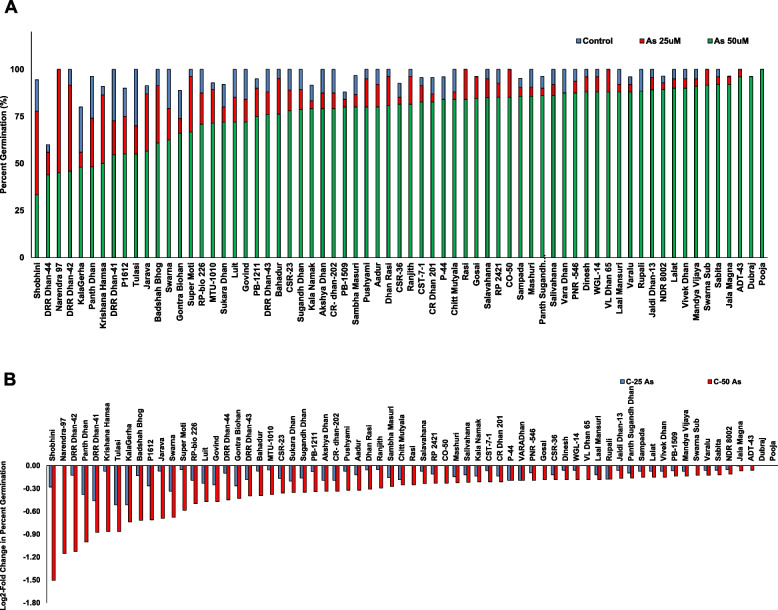
The result of **A** percent germination and **B** log2-fold change in % germination of genotypes for control versus As (III) stress of 67 rice genotypes

Rice genotypes reflected contrasting responses for germination and early seedling growth. For a particular genotype, responses to As(III) stress were also variable depending on As(III) concentration (25 µM and 50 µM). There were morphological differences (curling of leaf margins, wavy roots, variation in length and number of roots hairs) also in genotypes upon exposure to As(III) stress. A wide variation in percent germination was observed in control and As(III) stressed conditions. The germination percentage varied from 60%-100% in control, 56%-100% at 25 µM As(III) and 33–100% at 50 µM As(III) treatments (Fig. 1A). The log2-fold change in percent germination in control versus As(III) treatments was calculated to understand the response of genotypes. At 25 µMAs(III), except ADT-43, Dubraj and Pooja, all genotypes showed decrease in germination with the maximum reduction being -0.51-fold. At 50 µMAs(III), only Pooja and Dubraj were yet unaffected and showed 100% germination while other genotypes showed decline in germination ranging from -0.06-fold to -1.50-fold (Fig. 1B). Shobini showed the maximum decline in germination (-0.28 and -1.50-fold at 25 µM and 50 µM, respectively).

**Fig. 2:**
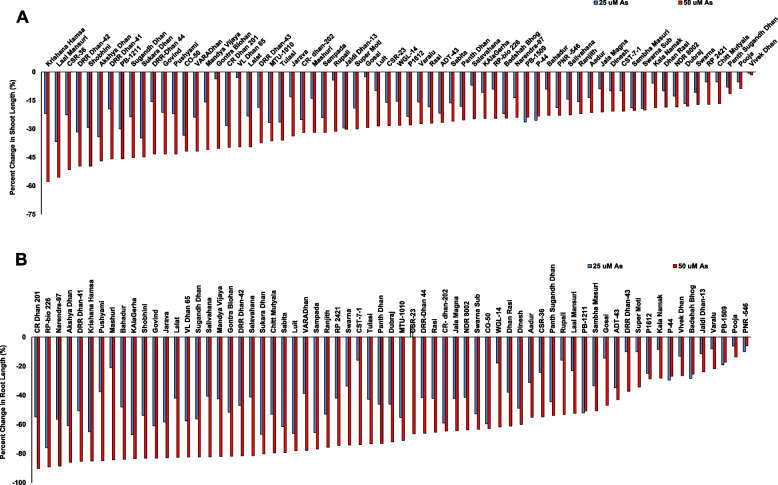
The results of percent change in **A** shoot length and **B** root length of 67 rice genotypes under As(III) stress. The percent change in shoot/root length was calculated for control versus As(III) stress at 25 and 50 μM As(III)

All the genotypes showed change in shoot growth upon As(III) stress (Supplementary Figure 1). The percent decline in shoot growth in As(III) as compared to control varied from 0.64% to 37% at 25 µM and from 1.63% to 58% at 50 µM. Vivek Dhan showed least negative effect of As(III) stress followed by Pooja, while Krishana Hamsa and Laal Mansuri showed the maximum decline in shoot growth (Fig. 2A). Similar to shoot growth, root growth was significantly affected by As(III) stress in all the genotypes (Supplementary Figure 2). The percent decline in root growth in As(III) treated seedlings as compared to control was found to be maximum 76% and 91% at 25 µM and 50 µM As(III) treatments, respectively. PNR-546 showed the least decline in root growth followed by Pooja while CR Dhan 201 showed the maximum decline in root growth (Fig. 2). As both shoot and root length varied greatly upon As(III) stress, the ratio of shoot/root length also showed a wide range of variation (Supplementary Figure 3). Hence, both germination and seedling growth showed a wide range of variation among genotyeps. To evaluate the results clearly, the seedling vigour index was used.

**Fig. 3:**
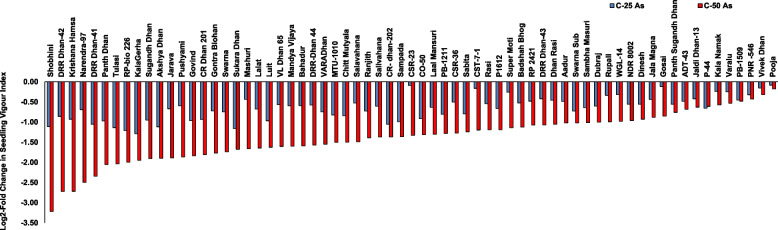
The result of log2-fold change in seedling vigour index of genotypes for control versus As (III) stress for 67 rice genotypes

**Fig. 4:**
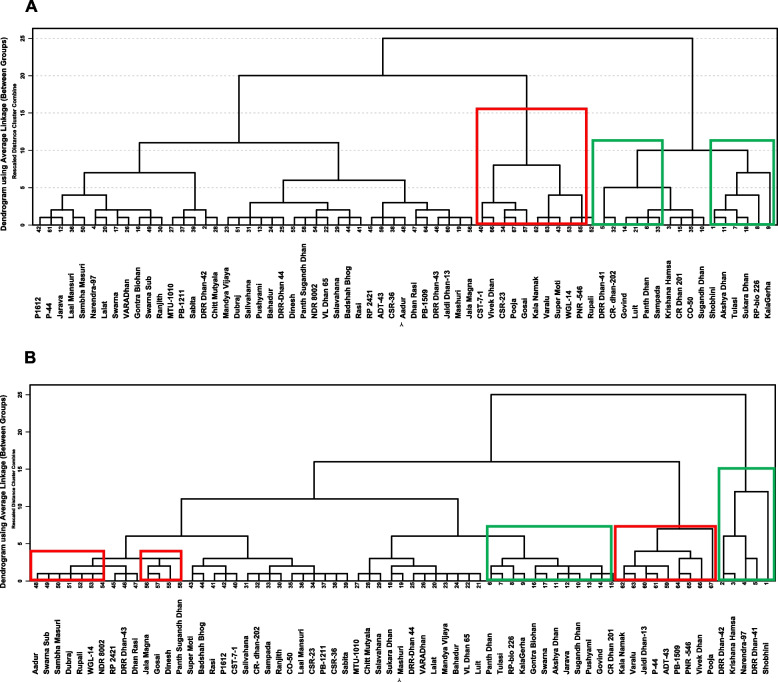
Dendrogram of the two desired classes, tolerant (marked red) and sensitive (marked green), assigned by the cluster analysis for 25 μM As(III) (**A**) and 50 μM As(III) (**B**)

The parameter of seedling vigour index was chosen to compare all the genotypes with respect to root and shoot growth and germination percentage. In control conditions, seedling vigour index varied from a minimum of 48 to a maximum of 273. Upon As(III) stress, seedling vigour index showed a decline with the range being 32–186 at 25 µM and 10–166 at 50 µM As(III (Supplementary Figure 4). Log2-fold change in seedling vigour index for control versus As(III) treatments was also calculated. It was found that Pooja showed the least decline in seedling vigour index upon As(III) treatment (-0.17-fold at 50 µM) while Shobhini showed the maximum decline seedling vigour index (-3.22-fold at 50 µM) (Fig. 3). Clustering analysis of genotypes was performed for seedling vigour index for the response of genotypes at 25 and 50 μM As(III) could group sensitive and tolerant genotypes (Fig. 4). Through these analyses, the tolerant genotypes like Pooja, VivekDhan, NDR8002, Kala Namak, PB-1509 Jaldi Dhan-3, and P-44, and sensitive genotypes like Shobhini, DRR Dhan-41, DRR Dhan-42, KrishanaHamsa, and Narendra-97 were identified.

Genotypic pool exists as one of the most important resources today. This is because genotypic variations are the source of important genes and properties that can be utilized to tackle several of the worlds’ environmental problems. However, yet most of these resources are not thoroughly evaluated to screen and select genotypes / varieties for a particular environmental condition. Several states of India have been reported to have As contamination and this contamination leads to As entry into crop plants; especially rice. Thus the identification of As tolerant genotypes of rice is a priority task. Some studies have been conducted previously in this direction and rice genotypes differing in As tolerance and seedling and grain As accumulation have been identified in India (Majumder et al. 2018; Dave et al. 2013; Dwivedi et al. 2012).

The results of screening clearly showed that genotypes differed greatly in their growth pattern even under control conditions. The exposure of genotypes to As(III) stress induced negative effects on germination and seedling growth that varied for different genotypes. A large variation in the response of genotypes was noticed. While some genotypes like Shobhini, DRR Dhan-44 showed more than 1-fold reduction in the germination at 50 μM As(III) in comparison to control, other genotypes like Pooja, Dubraj, ADT-43, and Jal Magna exhibited no change or only about 0.06-fold reduction in germination. Similarly, contrast responses of genotypes were noticed with respect to shoot and root length and the ratio of shoot/root length. The results of Log2-fold change in seedling vigour index also depicted reduction of more than 2.50-fold for Shobhini, DRR Dhan-42, Krishana Hamsa and Narendra-97 while less than 0.5-fold reduction for Pooja, Vivek Dhan, PNR-546 and PB1509. Clustering analysis of genotypes for seedling vigour index for the response of genotypes at 25 and 50 μM As(III) could group sensitive and tolerant genotypes in different groups. For further evaluation of responses of genotypes, two contrasting rice genotypes: one tolerant (Pooja) and one moderately sensitive (CO-50) were chosen.

### Duration and concentration dependent responses of contrasting rice genotypes to arsenic stress

#### Arsenic accumulation and growth

**Fig. 5:**
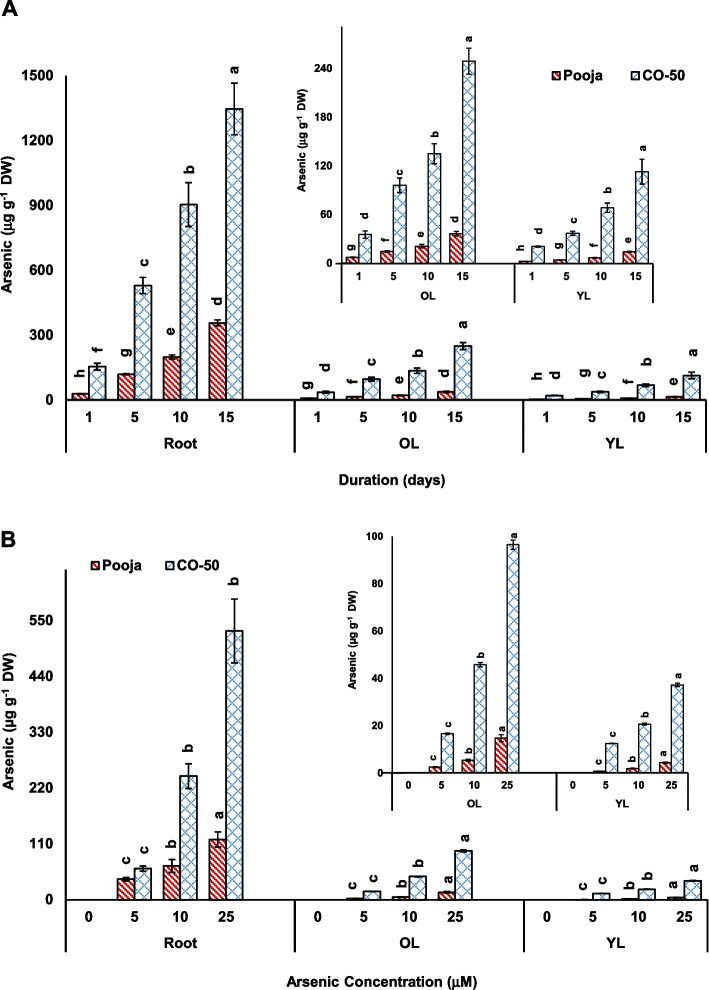
Arsenic accumulation in root, old leaves (OL) and young leaves (YL) of Pooja and CO-50 analyzed in response to different durations (1–15 d at fixed concentration of 25 μM) (**A**) and different concentrations (0–25 μM at fixed duration of 5 d) (**B**). The values are mean of three replicates ± SD with each replicate represented by 25 seedlings. ANOVA is significant at *p* ≤ 0.01. Different symbols on bars indicate significantly different values (DMRT; *p* ≤ 0.05)

In this study, As accumulation in different tissues was found to increase progressively in both concentration- and duration-dependent experiments. Pooja showed significantly lower As than CO50 in all treatment conditions and all tissues. In concentration-dependent experiment, the level of As in roots, old leaves (OL) and young leaves (YL) of Pooja was about 1.5–4.5-fold, 6.6–8.7-fold and 8.7–17.9-fold lower than that in CO-50. In duration-dependent experiment also, the difference in As content in Pooja and CO-50 was about 3.8–tenfold in various tissues. Hence, Pooja, the tolerant variety, showed consistently lower As accumulation in its tissues as compared to that of CO-50 (Fig. 5). A variable accumulation of any toxicant is a distinctive feature of a tolerant and a sensitive genotype of a plant; in this case As in rice (Chakrabarty et al. 2009). It is indeed the ability to resist the accumulation of As and also its translocation to above-ground tissues including new young leaves that imparts the tolerance to As to the genotype. In this work, it was clearly noticed that Pooja resisted As accumulation and its translocation to YL and this would have avoided excessive and rapid buildup of As in its tissues (Dave et al. 2013). In contrast, CO-50 would have faced rapid buildup of high amount of As that puzzled its protective mechanisms and sensitized it greatly to As toxicity (Yadav et al. 2021). The differential As accumulation allowed tolerant genotype Pooja to show better root and shoot growth (length and fresh weight) (Supplementary Figures 4 and 5). In contrast, CO-50 got exposed to high level of As and depicted greater toxicity in terms of growth of seedlings (Supplementary Figures 4 and 5). In concentration-dependent experiment, root lengh, shoot length and fresh weight showed 18%, 40% and 19% decline, respectively in Pooja in comparison to control while these parameters declined by 30%, 48% and 31%, respectively in CO-50. In duration-dependent set also, similar observations were made (Supplementary Figures 4 and 5). Hence, the growth of Pooja was positively attributable to lower As accumulation in its tissues while the opposite was true for CO-50.

#### Biochemical response of contrasting genotypes

To avoid toxicity, plants deploy two-way mechanism. Firstly they attempt to complex most of the As entering into cells and that too preferably in roots so as to reduce its translocation to shoot and its free ion concentration in the cell (Srivastava et al. 2007). This is considered as the primary strategy and the drivers for this are the thiol (-SH) containing ligands like glutathione (GSH) and phytochelatins (PCs), and cell wall (Raab et al. 2005; Srivastava et al. 2016). The second level of tolerance mechanism involves the mitigation of toxicity caused by free As ions that avoided complexation. The main players in this part are the antioxidants which prevent uncontrolled reactive oxygen species (ROS) generation and protect important biomolecules (Li et al. 2023). To further understand the tolerance mechanism of Pooja, oxidative stress was evaluated in terms of MDA content, antioxidant potential was tested by assaying SOD, the foremost antioxidant enzymes and the ability to complex As was determined by measuring total NP-SH levels.

**Fig. 6:**
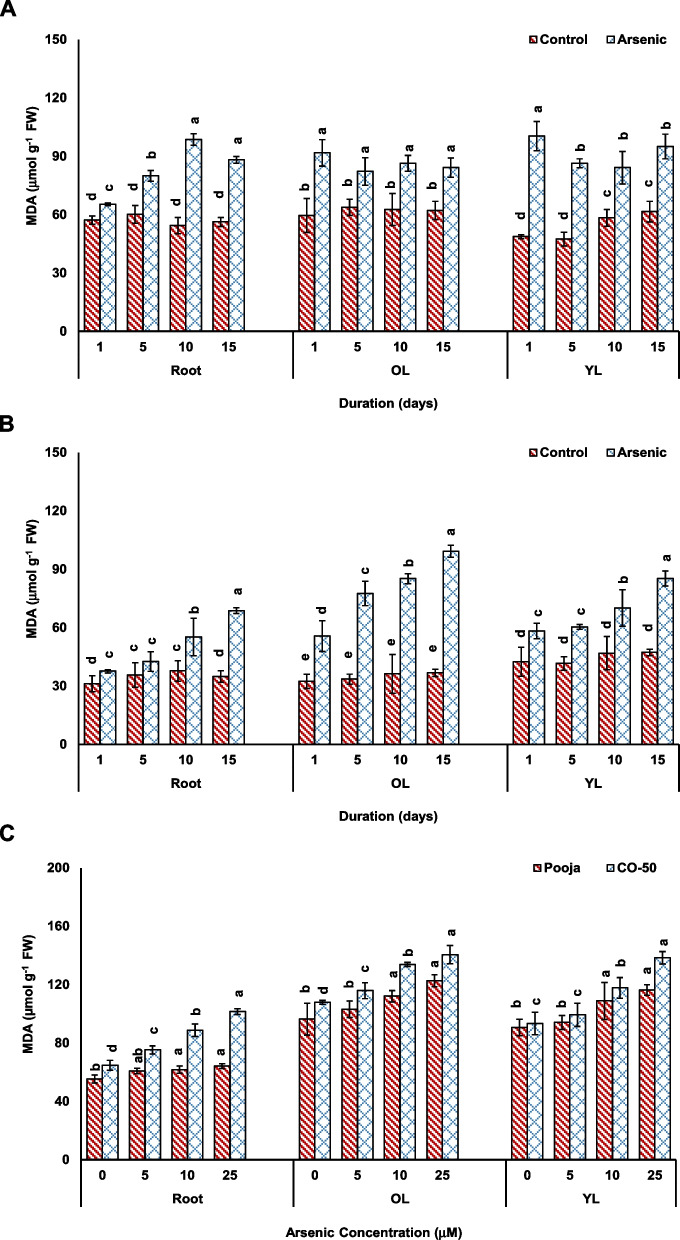
Effect of As stress on MDA levels in root, old leaves (OL) and young (YL) analyzed in response to different durations (1–15 d at fixed concentration of 25 μM) in Pooja (**A**) and CO-50 (**B**) and at different concentrations (0–25 μM at fixed duration of 5 d) (**C**). The values are mean of three replicates ± SD with each replicate represented by 25 seedlings. ANOVA is significant at *p* ≤ 0.01. Different symbols on bars indicate significantly different values (DMRT; *p* ≤ 0.05)

Pooja and CO50 showed different trends in MDA and SOD. MDA levels increased upon As exposure in concentration- and duration-dependent experiments. However, the increase was significantly higher in CO-50 than that in Pooja in all tissues (roots, OL and YL). In duration-dependent experiment, the increase in MDA level at 15 in root, OL and YL was 57%, 35% and 54%, respectively in Pooja and 97%, 170% and 80% in CO-50. In concentration-dependent experiment also, the increase in MDA in roots, OL and YL was 16%, 27% and 28%, respectively at 25 μM in Pooja, while in CO-50, the respective increases were 57%, 30% and 48% (Fig. 6). MDA is generated by peroxidation of lipids of membranes and the peroxidation process is initiated by ROS that in turn increase in response to a stress including As (Morales and Munne-Bosch 2019). Therefore, MDA variable increase indicated that CO-50 suffered from greater oxidative stress and subsequent damage to its lipid membranes than that of Pooja. Obviously, higher MDA level seems to be attributable to greater oxidative stress caused by more As accumulated in CO-50 than that in Pooja. The data of MDA analysis confirmed the differential sensitivity of the two varieties.

**Fig. 7:**
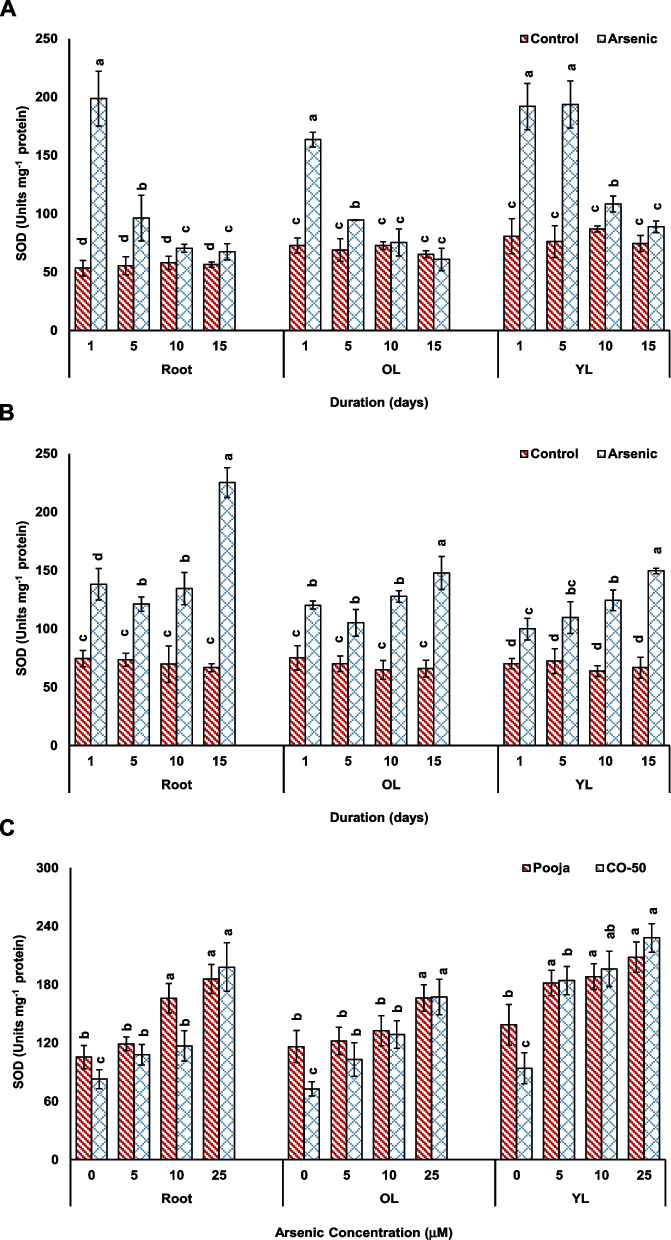
Effect of As stress on SOD activity in root, old leaves (OL) and young leaves (YL) analyzed in response to different durations (1–15 d at fixed concentration of 25 μM) in Pooja (**A**) and CO-50 (**B**) and at different concentrations (0–25 μM at fixed duration of 5 d) (**C**). The values are mean of three replicates ± SD with each replicate represented by 25 seedlings. ANOVA is significant at *p* ≤ 0.01. Different symbols on bars indicate significantly different values (DMRT; *p* ≤ 0.05)

Further, the activity of SOD affirmed the possible assumption of more ROS production in CO-50. The assay of SOD activity depicted that it increased significantly more in both concentration- and duration-dependent experiment in CO-50 than in Pooja (Fig. 7). The increases in CO-50 in roots, OL and YL at 25 μM were 140%, 130% and 142%, respectively that were more as compared to that seen in Pooja (76%, 43% and 50%). Similar response was noticed in duration-dependent experiment also. One peculiar observation in duration-dependent experiment was that in Pooja SOD activity showed greater increases at 1 d (272%, 124% and 138% in roots, OL and YL) and then the increase became lesser with the increase in duration (Fig. 7). In contrast, reverse trend was observed in CO-50 that depicted greater increase in SOD activity with increase in duration. This suggested that the initial sudden burst of As influx and consequent oxidative stress was aggressively tackled by Pooja than by CO-50. As seen in our earlier study, there are differences in perception of As stress in Pooja and CO-50 (Yadav et al. 2021). SOD is considered as the first line of defense against ROS since electrons leaking in disturbed redox reactions and electron transport chains reach to oxygen and firstly produce superoxide radicals (Chen et al. 2024; Mishra and Sharma 2019). Hence, an early response from SOD acts like a boon in the defense of plants against a stress. Pooja responded at an early time point and restricted As influx and consequently oxidative stress while CO-50 could not respond quick enough and continued to face incoming As and stress for prolonged duration that ultimately reflected in reduced growth.

**Fig. 8:**
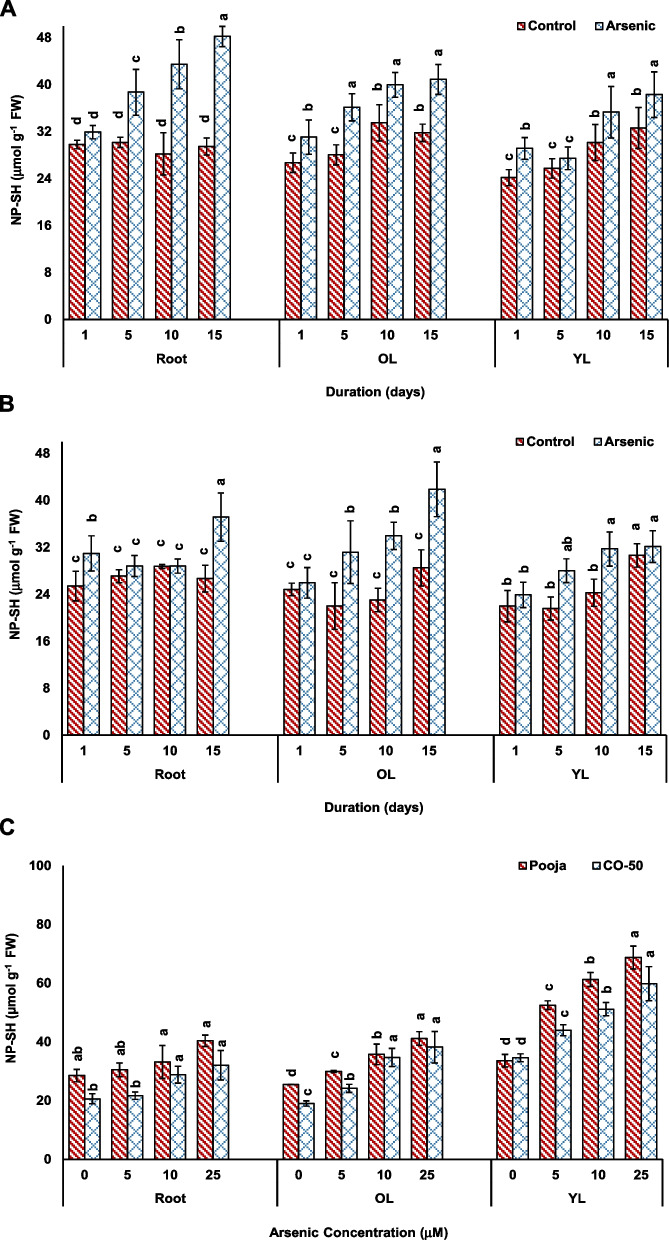
Effect of As stress on NP-SH level in root, old leaves (OL) and young leaves (YL) analyzed in response to different durations (1–15 d at fixed concentration of 25 μM) in Pooja (**A**) and CO-50 (**B**) and at different concentrations (0–25 μM at fixed duration of 5 d) (**C**). The values are mean of three replicates ± SD with each replicate represented by 25 seedlings. ANOVA is significant at *p* ≤ 0.01. Different symbols on bars indicate significantly different values (DMRT; *p* ≤ 0.05)

The assessment of NP-SH levels also demonstrated variable response in two genotypes. In concentration-dependent experiment, roots and OL showed greater increase in NP-SH in CO-50 than CO-50 at all concentrations while YL depicted higher increase in Pooja than in CO-50 (Fig. 8). In duration-dependent analysis also, the response of roots, OL and YL was mixed. Hence, both genotypes attempted to complex most of the As by synthesizing more thiols upon As entry. The increase in the synthesis of GSH and PCs at the expense of cysteine and great burden on sulfur metabolism is a feature of As-stressed plants (Srivastava et al. 2016). However, the variable response of Pooja and CO-50 can be visualized if we consider that correlation between NP-SH and As levels of two genotypes. One As molecule binds with three –SH molecules (Raab et al. 2005). The higher level of As in CO-50 will consume more NP-SH as compared to that in Pooja and therefore, in CO-50 normal functions of NP-SH, especially GSH, would be hampered. GSH takes part in numerous reactions in cell including redox balance and ROS quenching (Hasanuzzaman et al. 2017). An excessive consumption of GSH or a decline in the synthesis of GSH both have negative impacts on plant physiology (Hasanuzzaman et al. 2017). Hence, despite the increase in NP-SH appearing to be similar, CO-50 experienced greater stress than Pooja.

Rice genotype screening has been performed in previous studies both at lab and field scale also by various workers. In an earlier study, Murugaiyan et al. (2021) evaluated 53 rice genotypes on the basis of germination and seedling performance. They found significant differences among the genotypes in germination and growth and biochemical responses (chlorophyll, root and shoot length, and As content in seedlings). They found that some genotypes had significantly high As accumulation in shoot (M401 and TKM9) while others were shoot As excluders (NPT-IR68552-55-3-2, PSBRc82, and OM997). On the basis of As content and tolerance, genotypes NPTIR68552-55-3-2, OM997, Zhong413, and WTR1 (BRRI dhan69) were suggested to be highly tolerant, which restricted As accumulation in shoots. Rai et al. (2015) worked on low (CN1646-5, Nayanmoni and CN1646-2) and high (BRG-12, BRG-15, and BRG-20) arsenic accumulating rice genotypes and found that genotypes differ significantly in their transcriptomic responses to As stress. Gupta and Ahmad (2014) found that rice variety PB-1 could tolerate As stress through induction of antioxidants like cysteine, proline, superoxide dismutase, catalase and ascorbate peroxidase. The identification of suitable As-tolerant genotypes acts as a potential resource and the present study adds to that knowledge.

In conclusion, the present study identified potential tolerant and sensitive genotypes of rice towards As stress. The response of two contrasting genotypes was further assessed and it was found that variable As accumulation is one of the prime reasons for differential tolerance of two genotypes. The identified genotypes may act as potential genetic resource for varietal development programs for developing low arsenic accumulating varieties. Further, such contrasting genotypes might pave the way for identification of novel genes involved in As tolerance and accumulation that could be utilized in modern genetic approaches to generate low arsenic accumulating rice plants. The use of such As tolerant variety to get As-free rice grains would avoid costs associated with the use of any chemical /biological amendments for reducing As in rice. Hence, the tolerant genotype holds promise for practical application also in As contaminated fields.

## Materials and methods

### Collection of rice genotypes and arsenic exposure for screening

Rice genotypes were collected from Varanasi and Siddharth Nagar (Uttar Pradesh, India), Kolkata (West Bengal, India), Indian Council of Agricultural Research (ICAR)-Indian Institute of Rice Research (IIRR), Hyderabad, India and ICAR-Indian Agricultural Research Institute (IARI), New Delhi, India. A total of 67 rice genotypes were collected for the present study. Rice genotypes were grown hydroponically in a plant growth chamber (POL-EKO Aparatura-Make Climate Chamber with Phytotron Systems) under controlled conditions in As(III) (NaAsO_2,_ Sodium Meta-arsenite Sigma; 25 µM and 50 µM). The selection of As(III) doses was based on preliminary experiments with a few collected genotyeps to know the LC50 dose of As(III) and it was found to be in range of 21–26 µM and hence, a dose around LC50 and a higher dose were chosen for the experiments. For germination, seeds were sterilized with 30% ethanol and then soaked in water at 25ºC for 14 to 16 h by placing beakers on a shaker at ~100 rpm. The seeds were then spread on seed beds in Pertiplates and allowed to germinate in dark condition in the incubator (~48 h, 36 °C). After germination, seeds were grown in ½-strength Kimura B medium in dark and light period of 10 h and 14 h (260–350 μmol m^−2^ s^−1^ PAR) with temperature ranging from 23 °C to 28 °C. Thirty mL per seedling (5 seedlings per beaker) nutrient medium was insured in each beaker and medium was changed at alternate day in control and treatments sets. After 4 d growth in control conditions, beakers were divided into different sets: control, 25 µM As(III) and 50 µM As(III) and as per the division, were supplied with control or As(III)-containing medium. At 7d of stress exposure, plants were harvested and washed with milli-Q water. Root and shoot lengths were measured by metric scale.

### Analysis of germination percentage and seedling vigour index

For germination, 25 seeds of each cultivar were placed separately on a blotting paper in 9 cm diameter Petri plates covered with a lid and kept under incubation at 30 °C in dark for germination. Blotting papers were presoaked in ½ strength Kimura B medium for control and ½ strength Kimura B medium containing 25 µM As(III) and 50 µM As(III) for treatments. Total number of germinated seeds out of total seeds was recorded on the arrival of 2 mm or more of both plumule and radicle from the seed coat. Total number of germinated seeds was counted 3 d after incubation period and the results are expressed as percent change in comparison to control. The experiments were performed in triplicate set for both control and treatments.

Seedling vigour is not a single measurable property (like germination) but a concept describing several characteristics associated with seedling performance in the experimental conditions or field conditions (van de Venter 2001; Roberts and Osei-Bonsu 1988). Seedling vigour was calculated for each variety of rice by applying the following equation (Srinivas et al. 2016).

$$\text{Seedling Vigour Index }= (\text{MRL}+\text{MSL})*\text({\% \text G}/10)$$

Where, MRL = Mean root length; MSL = Mean shoot length; %G = percent germination

### Analysis of responses of contrasting rice genotypes: experimental conditions and assays

From the screening varieties, one tolerant and one moderately sensitive variety, namely Pooja and CO-50 were chosen. For various analyses, the two varieties were grown hydroponically in control conditions for 10 d as mentioned above. After 10 d, seedlings were exposed either to variable concentration of As(III) (0–25 μM) for fixed duration (5 d) or to a fixed As(III) concentration (25 μM) for variable duration (1 d to 15 d). After harvesting, root and shoot length were measured by metric scale and fresh weight was recorded after gently blotting the plants with a tissue paper. Plants were then separated into roots, old leaf (OL) and young leaf (YL). For various analyses, samples were homogenized in liquid nitrogen and stored at -80 °C until further use.

### Analysis of arsenic accumulation

Total As in the rice plant samples (root, OL and YL) was estimated after acid digestion of oven-dried plant samples (100 mg) in 1 mL of concentrated HNO_3_ on a heating block as described previously (Yadav et al. 2021).

### Assay of lipid peroxidation, superoxide dismutase activity and non-protein thiols

Lipid peroxidation was determined as 2-thiobarbituric acid (TBA) reactive metabolites chiefly malondialdehyde (MDA) accumulation (Heath and Packer 1968). Protein content in the enzyme extract was determined by following the Lowry et al. (1951). The assay of superoxide dismutase (SOD) activity was performed by following the method of Beauchamp and Fridovich (1971). The level of non-protein thiols (NP-SH) was measured following the method of Ellman (1959). The detailed of the methods were as described previously (Srivastava et al. 2006).

### Statistical analysis

The analyses were done in three replications for each treatment. Total 25 seedlings were used for each treatment. The results shown in graph are presented either as mean values ± SD and or as percentage change. Further, the results of control versus As(III) response of genotypes for different parameters was transformed to Log2 values for better comparison. Experimental data were analyzed statistically by using the SPSS 22 package for Cluster analysis for the seedling vigour. Data were analyzed by Analysis of variance (ANOVA) at *P* ≤ 0.01 and the means were statistically compared among treatments by the Duncan’s multiple range test (DMRT) at the *P* ≤ 0.05 level using the IBM SPSS 23 software.

## Supplementary Information

Supplementary Material 1.

## Data Availability

All data generated or analysed during this study are included in this published article [and its supplementary information files].

## Abbreviations

ANOVA: Analysis of variance
As: Arsenic
As(V): Arsenate
As(III): Arsenite
DMRT: Duncan’s multiple range test
MDA: Malondialdehyde
NP-SH: Non-protein thiols
SOD: Superoxide dismutase
