# Supplementary material for: Evaluation of the responses of contrasting sensitive and tolerant rice genotypes to arsenic stress
Source: Stress Biol. 2025 Mar 5;5(1):18. doi: 10.1007/s44154-024-00185-7 (PMC11880472; doi:10.1007/s44154-024-00185-7)
Supplement: Supplementary file 1 — Supplementary Material 1. [file 44154_2024_185_MOESM1_ESM.docx]

**Supplementary Information**

**Evaluation of the responses of contrasting sensitive and tolerant rice genotypes to arsenic stress**

Supplementary Figure 1: The result of shoot length for 67 rice genotypes. All the values of mean ± SD of 5 replicates.

Supplementary Figure 2: The result of root length for 67 rice genotypes. All the values of mean ± SD of 5 replicates.

Supplementary Figure 3: The result of ratio of shoot length / root length for 67 rice genotypes.

Supplementary Figure 4: The result of seedling vigour index for 67 rice genotypes.

Supplementary Figure 5: The effect of As(III) stress of 25 μM given for variable duration on root and shoot length of Pooja (A) and CO-50 (B) and fresh weight of whole seedlings (C). The values are mean of three replicates ±SD with each replicate represented by 25 seedlings. ANOVA is significant at *p ≤* 0.01. Different symbols on bars indicate significantly different values (DMRT; *p ≤* 0.05).

Supplementary Figure 6: The effect of variable concentrations of As(III) stress given for a fixed duration of 5 d on root and shoot length of Pooja and CO-50 (A) and fresh weight of whole seedlings (B). The values are mean of three replicates ±SD with each replicate represented by 25 seedlings. ANOVA is significant at *p ≤* 0.01. Different symbols on bars indicate significantly different values (DMRT; *p ≤* 0.05).
